# Emergent Completion Pneumonectomy for Postoperative Hemorrhage from Rupture of the Infected Pulmonary Artery in Lung Cancer Surgery

**DOI:** 10.1155/2011/902062

**Published:** 2011-07-31

**Authors:** Takanori Ayabe, Tetsuya M. Shimizu, Masaki Tomita, Mitsuhiro Yano, Kunihide Nakamura, Toshio Onitsuka

**Affiliations:** Department of Surgery II, Faculty of Medicine, University of Miyazaki, Kiyotake, Miyazaki 889-1692, Japan

## Abstract

Completion pneumonectomy (CP) is one of the most difficult procedures and known to be associated with a high morbidity and mortality. A 74-year-old male underwent a left upper lobectomy for pulmonary adenocarcinoma (T3N0M0); six days later after the surgery, he had a sudden postoperative intrathoracic excessive hemorrhage with shock. Emergent redo thoracotomy was performed to treat the bleeding from the ablated interlobar pulmonary artery by suturing with prolene. However, 3 days later after the second operation, he had the second intrathoracic bleeding. Emergent CP was performed with cardiopulmonary bypass by anterior transpericarsial approach via a median sternotomy. The hemorrhage was caused by a rupture of the proximal fragile and infected pulmonary artery. We performed omentopexy for the infected intrathoracic cavity and for covering of the divided main bronchial stump. We had a rare experience of two times of postoperative life-threatening hemorrhage from rupture of the infected pulmonary artery after left upper lobectomy. Emergent CP as salvage surgery should have an advantage in control of infected proximal pulmonary arterial hemorrhage. We should take care of tearing off of adventitia of pulmonary artery in lobectomy because of a possibility of postoperative hemorrhage under a fragility of the injured pulmonary artery with infection.

## 1. Introduction

Hemorrhage from the pulmonary artery in patient with lobectomy for lung cancer is a surgical emergency. Completion pneumonectomy (CP) is the removal of the lung or what is left of it from a previous ipsilateral lung resection. CP is one of the most difficult and unwelcome surgery for thoracic surgeons. We report a novel experience of two times of life-threatening intrathoracic hemorrhage with shock and a successful emergent CP under cardiopulmonary bypass, which was performed as salvage surgery for the postoperative bleeding due to the pulmonary arterial rupture with intrathoracic infection after left upper lobectomy for lung cancer. 

## 2. Case Report

**Figure 1:**
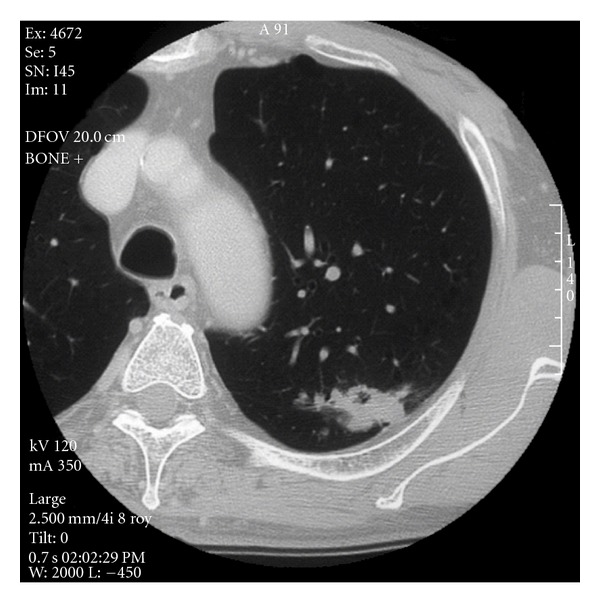
CT scanning of chest showing a lung cancer of 33 mm in the left S^1+2^.

A 74-year-old male with an abnormal shadow in the left upper field in the chest X-ray film. Computed tomographic scanning of chest showed a nodule of 33 mm in a diameter in the left S^1+2^ (Figure 1). He was diagnosed with adenocarcinoma of lung cancer by bronchoscopic examination (cT3N0M0, c-stage IIA). After posterolateral skin incision and 5th intercostal space thoracotomy, the lung was fibrously adhered to intrathoracic space. The incomplete interlobar pulmonary fissure was divided by GIA Stapling System (Blue, 45 mm, 3.5, COVIDIEN, Japan) with partial resection of lower lobe S6 region. GIA Stapling System (Gray, 45 mm, 2.0) was fired for dealing with A^1+2^ and A^3^ pulmonary arterial branches, and double of 2–0 silk were tied and cut for A^4a^, A^4b^, and A^5^ ones; GIA Stapling System (Blue, 45 mm, 3.5) was used for dividing of left upper bronchus. A left upper lobectomy with lymph nodal dissection was performed. The postoperative pathology resulted in adenocarcinoma with mixed subtype (acinar > papillary carcinoma, intralobar p3, br-, pa-, pv-, pT3N0M0, p-stage IIB). The continuous air leakage with low-grade fever had been observed until the 6th postoperative day (POD). At 14:30 on the 6th POD, a sudden intrathoracic hemorrhage with shock had been occurred. Emergent redo thoracotomy was performed to treat the excessive intrathoracic bleeding via the same operative incision. The hemorrhage was found from the exfoliated fragile lesion of interlobar pulmonary artery, which was closed with 5–0 prolene suture with pledgets. A large amount of intrathoracic hematoma had been removed. After the surgery, a continuous air leak with spiked fever had been continued until the 9th POD. The level of C-reactive protein was 28.6 mg/dL. At 19:50 on the 10th POD, sudden excessive hemorrhage (1500 mL) was occurred through thoracic drainage tube with shock. By pumping transfusion of red cell concentrates, the patient was moved to operating room; emergent salvage surgery was performed. By a median sternostomy approach and setting of cardiopulmonary bypass with cannulation into right femoral artery and vein, in the transpericardial manipulation, resections of main pulmonary artery with GIA Stapling System (White, 45 mm, 2.5) and superior and inferior pulmonary veins with GIA Stapling System (Gray, 45 mm, 2.0) were performed to quit the pulmonary arterial bleeding. Total blood loss was 4990 mL and 30 units of red cell concentrates and 16 units of fresh-frozen plasma were transfused. Although the thoracic cavity had been in the status of postoperative empyema thoracis, against where we used a pedicled omental flap to have a plompage effect, with which we covered to the divided left main bronchial stump. The median sternotomic wound had been closed; however, the left posterolateral chest wall with the 5th intercostal space had been open in a desire for an effective drainage and intrathoracic washing with saline via thoracic tube. *Staphylococcus aureus* was later proven by intrathoracic lavage culture. The patient had been stayed under three-week respirator control.

**Figure 2:**
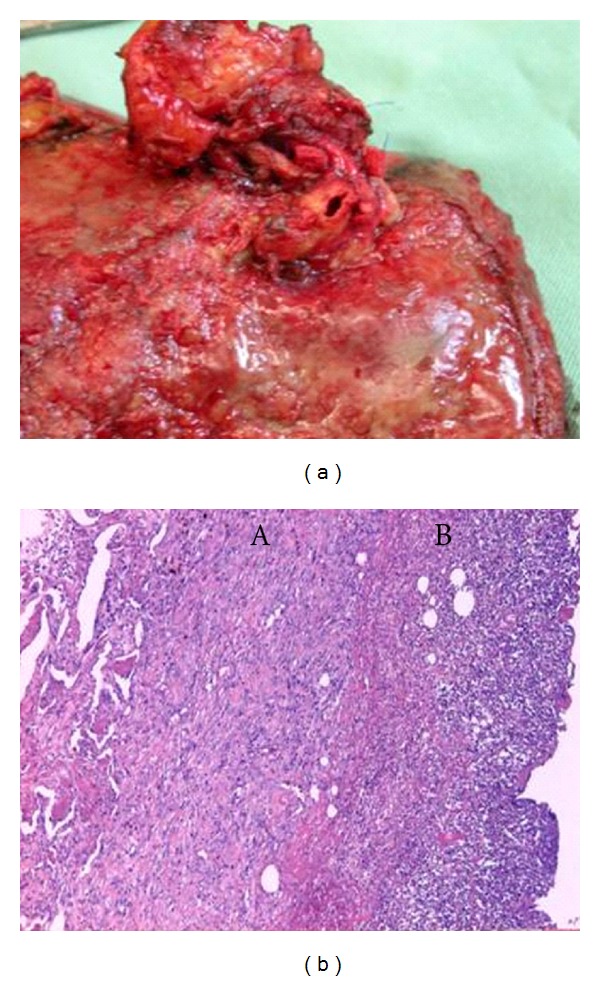
(a) Extensive inflammation of the resected lower lobe. (b) Lung parenchyma, B: pleura. Suppurative pan-pleuritis.

**Figure 3:**
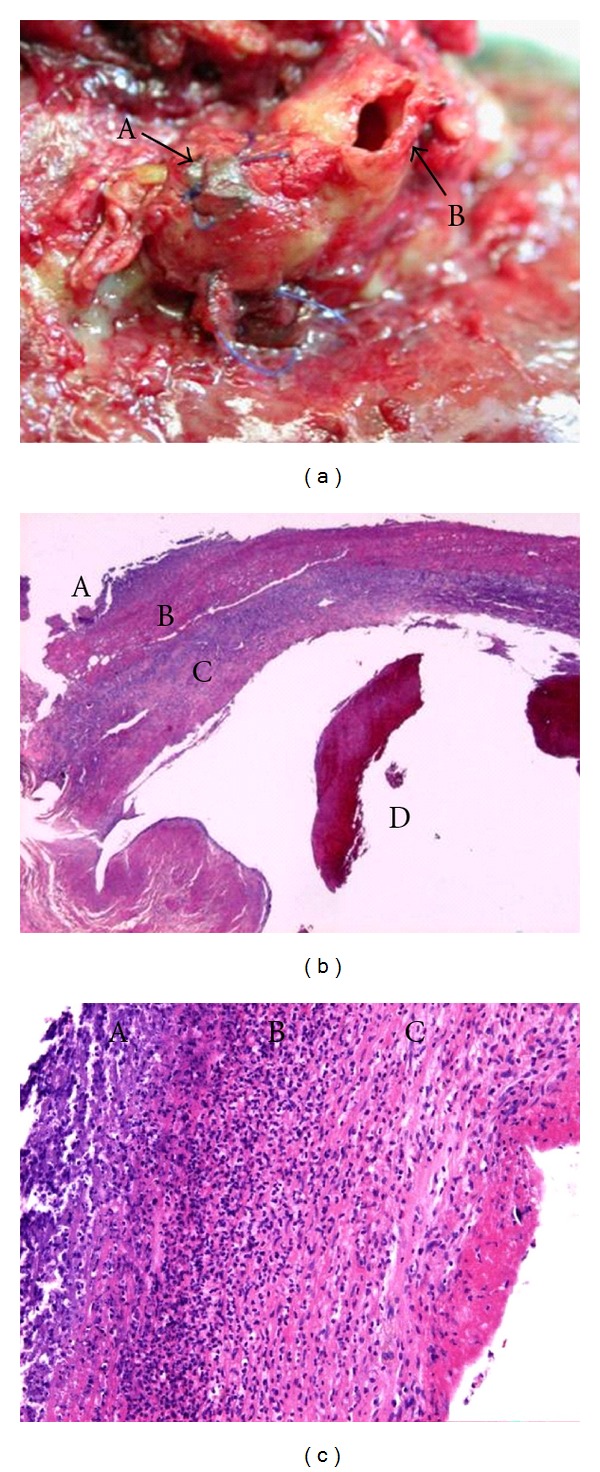
(a) The pulmonary artery was inflammated, edematous, and fragile. A: The first hemorrhage part in peripheral had been completely closed by prolene with pledgets. B: The second bleeding lesion was in the proximal lesion; a large cleft was opened. (b) A: adventitia; B: media; C: intima; D: organizing thrombi. Pathology disclosing the ruptured lesion of pulmonary arterial wall, in which adventitia and media tunicas were thin because of tearing off. (c) A: adventitia; B: media; C: intima. A severe inflammatory change has been observed in the three tunicas of the pulmonary arterial vessel.

The resected specimen revealed the inflammatory surface of lower lobe with extensive suppurative pleuritis (Figures 2(a) and 2(b)). The completely sutured lesion for the first bleeding displayed no abnormality; however, the second hemorrhage occurred in a different lesion, proximal pulmonary artery, and which resulted in a rupture due to infection (Figures 3(a) and 3(b)). Pathologically, there was a suppurative pan-pleuritis and disruption of pulmonary artery, and the pulmonary arterial wall was partly necrotic and marked infiltration of neutrophile was seen (Figures 2(b), 3(b) and 3(c)). Organizing thrombi were attached in the inflammated wall (Figure 3(b)). Histopathology resulted in adenocarcinoma with mixed subtypes and in no evidence of metastasis in lymph nodes. Tracheostomy was performed on the 26th POD. Respirator could have been weaned on the 29th POD. Two times a day of intrathoracic saline-washing had been continued for 84 days. The opened chest wall wound was closed on the 110th POD. The skin wound had been closed and he had been discharged on the 141th POD. He has been alive with no recurrence for seven years. 

## 3. Discussion

CP is an infrequent procedure and defined as below [1]. CP is the operation during which the remainder of a lung is removed, after one or more ipsilateral resections of lung parenchyma. A single-stage operation pneumonectomy is the operation in which an entire lung is removed without previous ipsilateral resection of lung tissue. The primary operation was defined as the first ipsilateral operation in which lung tissue was removed.

On CP in patients with recurrence or second primary cancer in the remained lung after surgery of primary lung malignancy, the perioperative complications rate was reported to be 30 to 70%, in which mortality rate was high to be 3.4 to 24% [2–7]. These were closely related with initial operative procedure and adjuvant treatment of chemotherapy and chemoradiotherapy, and patient's cardiopulmonary function with performance status. In the initial operation, manipulating of pulmonary artery and vein under incision of mediastinal pleura, which influenced to the hilum vessels and bronchus in mediastinum, as a resulted in severe adhesion and tissue fragility, it made the operation of CP difficult. Especially, tearing off of the adhesion perimembraneous pulmonary vessels had a high intraoperative lethal complication such as bleeding from injuring of proximal pulmonary artery. Intrapericardial manipulation of pulmonary vessels was reported to be safe and effective in case of the difficulty of tearing off of pulmonary vessels [1]. 

CP due to early complication of a first resection is a different operation from CP performed months or years after a first resection [8]. Emergency or urgent CP have to be performed in indication for a complications after the initial lung resection, which carries a higher risk of mortality than a standard operation. Jungraithmayr et al. [5] reported the indication of CP for complications for emergency and urgent condition as below. The emergency operation refers to the prompt reoperation in patients who had an unstable respiratory or circulatory status, such as bronchopleural fistula, infarction of the lung, empyema, and haematothorax. In contrast, the indication of urgency allows careful preparation and amelioration of the general condition prior to CP, such as bronchopleural fistula, empyema, infarction of the lung, destroyed lung, bronchial stenosis, and aspergilloma.

We surgeon sometimes had experienced disgraceful redo thoracotomy for intrathoracic bleeding after lobectomy in a few days of early complication. We had a very rare case of postoperative hemorrhage in several days after lobectomy, which might cause a lethal results. We suggested possibility of injuring of pulmonary arterial wall during the tearing off of interlobar pulmonary artery in the first operation and a complication of infection under continuous bronchioalveolar air leakage with fever. An intrathoracic bacterial infection had influenced the injured fragile part of the ablated pulmonary artery, which resulted in sudden hemorrhage as a rupture of pulmonary one. To quit the pulmonary bleeding and to close the ruptured pulmonary arterial wall, redo surgery had to be emergently performed with the same thoracotomic approach. However, for the second bleeding, emergent CP as salvage surgery should be performed under cardiopulmonary bypass via median full sternostomy and transpericardial approach in order to divide a more proximal, noninflammatory, nonadhesion, and normal pulmonary arterial wall. Simultaneously, omentoplasty would be performed to improve intrathoracic infection and to prevent a bronchopleural fistula. Open thoracic drainage after CP would bring an effective management of empyema.

## Abbreviation

CP: completion pneumonectomy.
